# Bioactive compounds intake in the Brazilian population: Trends and determinants of socioeconomic inequalities between 2008 and 2018

**DOI:** 10.1371/journal.pone.0292006

**Published:** 2023-10-05

**Authors:** Renata A. Carnauba, Flavia M. Sarti, Neuza M. A. Hassimotto, Franco M. Lajolo

**Affiliations:** 1 Department of Food Science and Experimental Nutrition, School of Pharmaceutical Sciences, University of São Paulo, São Paulo, Brazil; 2 Food Research Center, CEPID-FAPESP (Research Innovation and Dissemination Centers, São Paulo Research Foundation), São Paulo, Brazil; 3 Center for Research in Complex Systems Modeling, School of Arts, Sciences and Humanities, University of São Paulo, São Paulo, Brazil; Wroclaw University of Environmental and Life Sciences: Uniwersytet Przyrodniczy we Wroclawiu, POLAND

## Abstract

**Objective:**

The present study aims at evaluating trends and determinants of socioeconomic inequalities in consumption of bioactive compounds in representative sample of the Brazilian population the period from 2008–2009 to 2017–2018.

**Methods:**

Data from two cross-sectional population-based surveys were analyzed in the study, using descriptive analysis and estimation of inequalities in consumption. Trends in polyphenol and carotenoid intake were estimated using food consumption data from National Dietary Survey (NDS) 2008–2009 (n = 34,003) and 2017–2018 (n = 46,164). Evolution and determinants of inequalities in bioactive compounds intake were identified using analysis of inequality based on concentration index.

**Results:**

Consumption of total polyphenols, phenolic acids, flavonoids and carotenoid classes (except for zeaxanthin) was significantly associated with per capita income after adjustment for potential confounders, being higher income associated with higher intake of bioactive compounds. Disaggregation of inequalities showed that education was the main factor associated with consumption of flavonoids, other polyphenols and *β*-cryptoxanthin in 2008–2009; whilst income was the main barrier to intake of polyphenols and carotenoids in 2017–2018.

**Conclusion:**

Income level and educational attainment have been important determinants to inequalities in bioactive compounds intake in the Brazilian population throughout the period of analysis, being higher intake of bioactive compounds-rich foods and beverages associated with higher income.

## Introduction

Socioeconomic status represent important determinant of dietary intake, particularly in low- and middle-income countries marked by high inequalities like Brazil. Studies in diverse countries showed that individuals with higher income usually consume higher-quality diets with greater amounts of fruits and vegetables, and fewer sugar-sweetened beverages, than individuals with lower income [1–5]. In addition, our previous investigations showed that income level influenced dietary intake of bioactive compounds in 2008–2009, indicating higher consumption among Brazilian individuals with higher income in comparison to their counterparts [6, 7].

Bioactive compounds comprise a large and complex group of plant secondary metabolites, being widespread in plant-based foods and beverages [8, 9]. There is an increasing interest in health effects of bioactive compounds during the last decade, since several studies have showed inverse associations between their intake and the risk of chronic diseases, e.g., cardiovascular diseases (CVD) [10–12], type 2 diabetes [13, 14], and neurodegenerative disease [15, 16]. Considering the importance of bioactive compounds intake to health maintenance, it is important to understand long-term trends and inequalities in their consumption according to socioeconomic status, allowing the implementation of public health interventions and identification of potential intervention targets.

To date, studies have explored the socioeconomic and demographic factors related to food consumption and nutrient intake, with income, educational attainment and race being the main characteristics associated with access to healthy foods [4, 17–20]. In Brazil, previous research that analyzed trends of social inequalities in food consumption between 2008 and 2019 showed an increase in disparities in fruits and vegetables intake across levels of education [21]. Nevertheless, there is lack of evidence on the evolution and factors associated with socioeconomic inequalities in bioactive compounds intake in the Brazilian population. Thus, the aim of the present study was to evaluate trends and determinants of socioeconomic inequalities in consumption of bioactive compounds in representative sample of the Brazilian population the period from 2008–2009 to 2017–2018.

## Materials and methods

### Study population

Data of the present study were retrieved from the National Dietary Survey (NDS) 2008–2009 and 2017–2018, a cross-sectional survey conducted along with the Brazilian Household Budget Survey (HBS), which comprises a nationwide survey designed to determine the pattern of consumption and expenditures of the Brazilian population. The HBS adopted complex sample design, using two-stage cluster sampling with random selection of census tracts according to their geographical location and socioeconomic status. Detailed description on sampling procedures is available on previous publications of the Brazilian Institute for Geography and Statistics [22, 23]. Individuals within strata selected for data collection were evaluated over 12-month period to ensure representativeness and capture differences in seasonality during the year. The data collection of 2008–2009 NDS started on May 19^th^ 2008 and ended on May 18^th^ 2009. For 2017–2018 NDS, data collection occurred from July 11^th^ 2017 to July 9^th^ 2018.

In the 2008–2009 NDS, a random subsample of 13 596 (24.3%) households included in the HBS edition were assessed for individual food intake, corresponding to 34 003 subjects ≥10 years old. In the 2017–2018 NDS, individual food intake was collected in a random subsample of 20 112 (34.7%) households included in the HBS, encompassing a sample of 46 164 subjects ≥10 years old.

### Dietary intake

In the 2008–2009 NDS, individual food intake data were collected from two non-consecutive dietary records. Participants were instructed to register detailed information on foods and beverages consumed throughout the day, including portion sizes, cooking methods, time and place of meals (at home or away from home). Trained researchers reviewed the dietary records along with participants, registering missing or incomplete information if necessary and typing the data into a digital database created for the survey.

In the 2017–2018 NDS, individual food consumption data were obtained through two non-consecutive 24-hour dietary recalls (24HR). Trained researchers conducted personal interviews following sequential stages based on the U.S. Department of Agriculture Automated Multiple-Pass Method [24]. Participants were asked about foods and beverages consumed on the previous day, and information on recipes, portion sizes, cooking methods, time and place of meals were recorded during the interview.

In order to provide reliable data on dietary bioactive compounds intake, recipes mentioned in the two NDS editions were converted into ingredients to estimate the amount of ingredients in mixed dishes. Daily intake of bioactive compounds was estimated based on the two dietary records (2008–2009 NDS) and the two 24HR (2017–2018 NDS).

### Food composition database and estimation of intake on bioactive compounds

**Fig 1:**
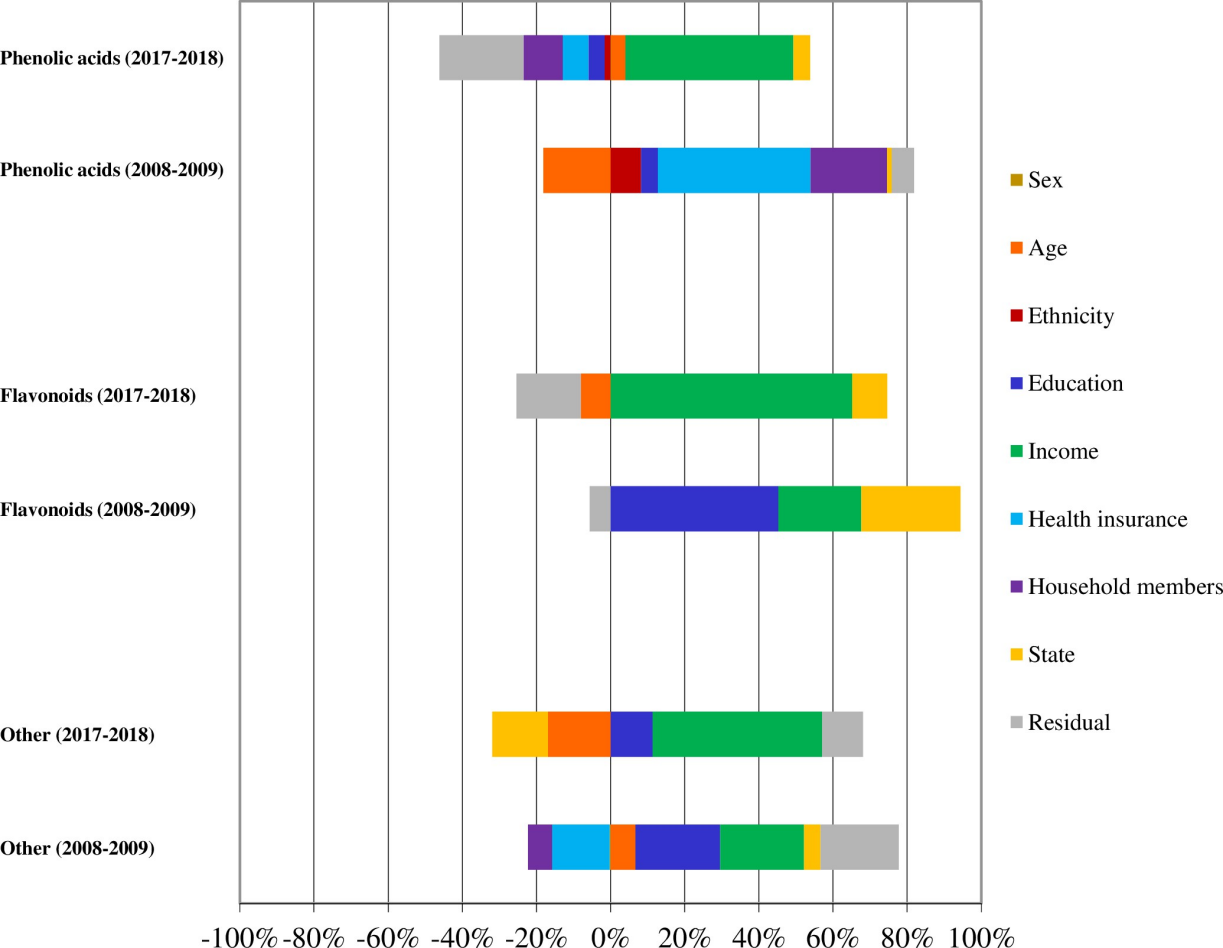
Chemical structure of the four major classes of polyphenols.

The Phenol-Explorer database (www.phenol-explorer.eu/) was used to retrieve the contents of polyphenols (Fig 1) in foods consumed by individuals interviewed in the two NDS editions. Data on the majority of polyphenols were determined by reverse-phase high-performance liquid chromatography (HPLC), except for proanthocyanins for which normal phase HPLC were used. Foods containing polyphenols linked to the food matrix that cannot be released under normal extraction conditions (e.g., lignans, ellagic acid in walnuts and hydroxycinnamic acids in cereals, beans and olives), data corresponding to HPLC after basic or acid hydrolysis were considered [25].

In addition to the Phenol-Explorer, regional foods and beverages (e.g., açai and yerba mate tea) and other items highly consumed by the Brazilian population (e.g., rice, beans and orange), we used data from foods collected and analyzed in Brazil through HPLC, and information available in the Brazilian Food Composition Database (TBCA, available in www.tbca.net.br/), an online database with data on flavonoid content (expressed as aglycone) identified and analyzed using HPLC [26]. In the case of overlapping data between Brazilian data and Phenol-Explorer database, Brazilian data had priority. Cooked foods reported in NDS dietary collection data, retention factors (RF) from Phenol-Explorer database were applied to allow accurate measurements of polyphenol intake [27].

Polyphenol intake was calculated as aglycone equivalents for foods containing polyphenols in forms of glycosides and esters, by removing the contribution to molecular weight of the non-phenolic part of the molecule for each polyphenol.

**Fig 2:**
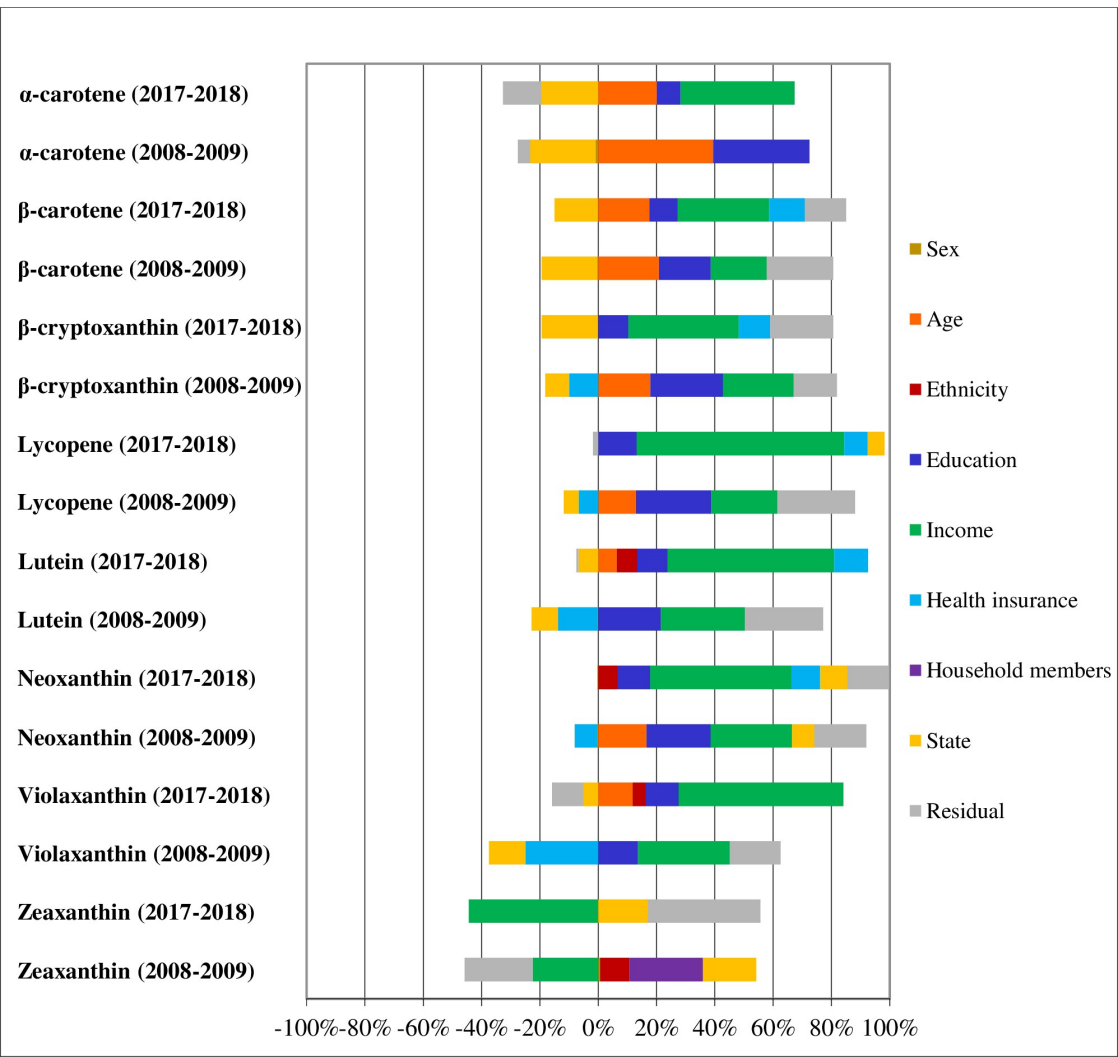
Disaggregation of income-related inequality in carotenoid intake. Brazil, 2008–2009 and 2017–2018.

Carotenoid concentration (*α*-carotene, *β*-carotene, *β*-cryptoxanthin, lycopene, lutein, neoxanthin, violaxanthin and zeaxanthin, Fig 2) of foods was obtained from the literature. The priority was to use data from foods harvested and analyzed in Brazil by high performance liquid chromatography (HPLC). We also used data from TBCA, which contains data on carotenoid content of Brazilian foods analyzed by HPLC [26].

In the case in which the carotenoid composition was not available for a food collected in Brazil, the data were obtained from HPLC analysis from Spain (avocado, asparagus, beetroot, onion, and apricot), Argentina (chard), China (persimmon), Indonesia (cashew nut), Iran (saffron), Italy (grape), Mexico (egg) and Portugal (cherry). Information collected on carotenoid concentrations corresponded to raw or cooked foods, depending on consumption characteristics described by interviewees.

The daily intake of polyphenols and carotenoids was estimated based on the two dietary records (2008–2009 NDS) and the two 24-h dietary recalls (2017–2018 NDS), using the same food composition data. Polyphenols and carotenoids intake from each food was calculated by multiplying contents by daily consumption amount of each food and dividing by 100, considering that food composition data is reported in mg/100 g. Total polyphenols and carotenoids intake was calculated by summing up intakes from all foods consumed.

### Socioeconomic and demographic characteristics

NDS interviews were conducted by trained researchers using a structured questionnaire including socioeconomic and demographic characteristics of individuals, and supplementary information on households (e.g., household members, ownership of goods, etc.). Information on household income included monetary and nonmonetary sources of household revenues (including donations and participation in income transfer programs), which were converted into monthly household income per capita by dividing household income by household residents. In order to ensure appropriate comparison of monetary values between periods, household income per capita from individuals interviewed in 2008–2009 were updated to 2017–2018 using the Brazilian Broad Consumer Price Index (Índice de Preços ao Consumidor Amplo, IPCA) from the Brazilian Institute for Geography and Statistics. Monetary values were subsequently converted into international monetary units using purchase power parity conversion factors to the reference data of 2017, based on information available at the World Bank platform, to allow comparison with data from other countries.

### Statistical analysis

Data were presented in median for continuous variables, and frequencies or percentages for categorical variables. Polyphenol and carotenoid intake data from the two dietary records (2008–2009 NDS) and the two 24HR (2017–2018 NDS) were statistically adjusted for the usual intake distribution and removal of intrapersonal variation using the statistical technique Multiple Source Method. Logistic regression was performed to explore the associations between compounds intake and per capita income.

Estimation of evolution and determinants of socioeconomic inequalities in the consumption of bioactive compounds were based on the concentration indexes according to type of compound and year of the survey, following Almeida and Sarti (2013) [28]. The concentration index measure inequality according to socioeconomic position of individuals (Eq 1).


$$CI=\frac{2}{n\mu }\sum_{i=1}^{N}y_{i}R_{i}-1$$ (1)

Being: *μ* = mean of *y* (consumption of polyphenols); *R*_*i*_ = fractional rank of *i*^th^. individual in income distribution; and *N* = sample size. Therefore, it is possible to measure inequality among groups of individuals with different personal characteristics through the horizontal inequality index (*HI*) by calculating differences between concentration indexes (*CI*) and concentration indexes controlled by individual characteristics (*CN*).

The method allows disaggregation of the concentration index according to main determinants, showing inequalities attributable to two dimensions: individual characteristics and external factors. The contribution of external factors allows to indicate features from the individuals’ environment that influence polyphenols consumption (e.g., socioeconomic background, regional characteristics, etc.), whilst individual characteristics identify individuals’ characteristics (sex, age, and skin color/ethnicity) contributing to inequalities. *CI* disaggregation is performed using linear regression models with dependent variable represented by the concentration index, y* (Eq 2).


$$y^{*}=\beta_{1}^{\prime }.X+\beta_{2}^{\prime }.Z+\varepsilon$$ (2)

Being: *βk* = coefficient of explicative variable *k*; *X* = matrix of variables of individual characteristics (sex, age, skin color/ethnicity); *Z* = matrix of variables of external factors (socioeconomic features, household characteristics, and geographical location variables); and *ε* = error term.

Analyses were performed using Stata software version 17.0, considering the complex sampling design, and p values <0.05 were considered significant.

## Results

Information on 34 003 participants from the 2008–2009 NDS and 46 164 individuals from the 2017–2018 were available for analysis. The associations between polyphenol intake and income in the 2008–2009 and 2017–2018 NDS are presented in Table 1. Total polyphenols, phenolic acids and flavonoids intakes were significantly associated with income after adjustment for sex, age, ethnicity, Brazilian region, area of residence, educational level, and energy intake (p≤0.0070) in both NDS editions.

**Table 1:** Associations between polyphenols* and carotenoids intake in relation to per capita income. Brazil, 2008–2009 and 2017–2018.

Bioactive compounds	2008–2009				2017–2018			
	Per capita income		P value		Per capita income		P value	
	Lowest	Highest	Unadjusted	Adjusted*	Lowest	Highest	Unadjusted	Adjusted**
Polyphenols								
Phenolic acids (mg/d)	113.9	348.9	0.0001	0.0001	112.5	350.7	0.0142	0.0070
Hydroxybenzoic acids (mg/d)	0.6	1.7	0.0001	0.0001	1.2	8.2	0.0001	0.0001
Hydroxycinammic acids (mg/d)	113.1	348.1	0.0001	0.0001	109.0	346.7	0.0705	0.0020
Flavonoids (mg/d)	47.0	1558.5	0.0001	0.0001	62.4	1162.2	0.0001	0.0001
Flavan-3-ols (mg/d)	3.5	67.1	0.0001	0.0001	3.3	57.8	0.0001	0.0001
Flavones (mg/d)	4.4	10.7	0.0001	0.9380	5.6	12.3	0.0010	0.1760
Flavonols (mg/d)	2.2	10.6	0.0001	0.0001	13.2	33.9	0.0001	0.0001
Flavanones (mg/d)	9.3	1487.5	0.0001	0.0001	8.5	1092.4	0.0001	0.0020
Anthocyanins (mg/d)	0.02	2.3	0.0001	0.0001	0.7	12.6	0.0001	0.0001
Isoflavonoids (mg/d)	0.0	0.2	0.0215	0.0001	0.0	0.1	0.0001	0.0001
Other (mg/d)	2.3	9.4	0.0001	0.0001	5.8	50.3	0.0001	0.0001
Total polyphenols (mg/d)	271.0	1740.6	0.0001	0.0001	272.3	1366.3	0.0001	0.0001
Carotenoids								
*α*-carotene (mg/d)	0.7	9.4	0.0001	0.0001	0.7	6.1	0.0001	0.0001
*β*-carotene (mg/d)	4.1	34.8	0.0001	0.0001	5.0	30.5	0.0001	0.0001
*β*-cryptoxanthin (mg/d)	0.1	0.5	0.0001	0.0001	0.1	0.7	0.0001	0.0001
Lycopene (mg/d)	0.9	5.5	0.0001	0.0001	1.5	6.9	0.0001	0.0001
Lutein (mg/d)	1.7	5.3	0.0001	0.0001	2.5	10.3	0.0001	0.0001
Neoxanthin (mg/d)	0.1	1.1	0.0001	0.0001	0.3	1.9	0.0001	0.0001
Violaxanthin (mg/d)	0.5	3.1	0.0001	0.0001	0.8	4.6	0.0001	0.0001
Zeaxanthin (mg/d)	0.4	1.1	0.1106	0.2811	0.4	1.1	0.1265	0.5999
Total carotenoids (mg/d)	12.4	60.5	0.0001	0.0001	15.8	60.1	0.0001	0.0001

(*) Aglycone equivalents.

(**) Adjustment for sex, age, ethnicity, Brazilian region, area (urban or rural), educational level, and energy and fiber intake.

Phenolic acids were the most consumed polyphenols class in the lowest income group, despite of slight decrease in consumption from 2008–2009 to 2017–2018. The contribution of flavonoids to total polyphenol intake increases according to increase in income, although it also showed small reduction over the period, being the main polyphenol class consumed by individuals in the highest income category. Consumption of phenolic acids and flavonoid subclasses by individuals in the highest income level showed significant differences (p≤0.0020) compared with individuals in the lowest income category, except for flavones.

Total carotenoid intake and classes (with exception of zeaxanthin) were significantly associated with income after adjustment for potential confounders (p<0.0001) in both NDS editions. *β*-carotene was the most consumed carotenoid class in the both income groups and NDS editions, followed by lutein and lycopene.

The three major food sources of total, classes and subclasses of polyphenols in 2017–2018 NDS are shown in Table 2. Coffee was the food item that most contributed to total polyphenols, phenolic acids and hydroxycinnamic acids intake, although the percentage of contribution decreases according to increases in income. The contribution of beans and preparations to total polyphenols, total flavonoids, flavonols and anthocyanins also decreases in the highest income group compared with the lowest income category. The intake of total flavonoids, flavanones and flavan-3-ols from orange, tea and salads increases with income, similarly to the contribution of alcoholic beverages (beer and wine) to hydroxybenzoic acids and flavan-3-ols intake. On the other side, the contribution of wheat flour products (bread, pasta and cracker) to flavones and other polyphenols intake decreases with the increase of income.

**Table 2:** Comparison of the main food sources of polyphenols according to per capita income in Brazil (2017–2018).

Polyphenol classes and subclasses	Rank	Lowest per capita income		Highest per capita income	
		Food item	%	Food item	%
Phenolic acids	1	Coffee	89.3	Coffee	81.1
	2	Rice and preparations	3.9	Rice and preparations	3.1
	3	Bread	1.5	Bread	0.7
Hydroxybenzoic acids	1	Tea	20.8	Tea	33.2
	2	Beer	9.8	Beer	14.3
	3	Rice and preparations	8.2	Wine	10.5
Hydroxycinnamic acids	1	Coffee	90.3	Coffee	83.2
	2	Rice and preparations	3.9	Rice and preparations	3.1
	3	Bread	1.5	Bread	0.7
Flavonoids	1	Bean and preparations	49.5	Bean and preparations	29.5
	2	Orange juice	12.6	Orange juice	15.0
	3	Tea	3.7	Tea	6.6
Flavan-3-ols	1	Tea	20.1	Tea	30.3
	2	Chocolate	8.0	Wine	16.9
	3	Chocolate powder	6.3	Chocolate	9.3
Flavones	1	Bread	39.1	Bread	36.6
	2	Pasta	25.3	Pasta	22.1
	3	Cracker	6.0	Cracker	3.5
Flavonols	1	Bean and preparations	60.2	Bean and preparations	36.4
	2	Salads	6.6	Salads	12.2
	3	Apple	5.3	Apple	6.5
Flavanones	1	Orange juice	83.7	Orange juice	79.6
	2	Orange	12.6	Orange	16.1
	3	Lemon	0.5	Fruit salad	1.5
Anthocyanins	1	Bean and preparations	19.8	Grape juice	16.9
	2	Açai	21.3	Açai	15.9
	3	Grape juice	10.1	Bean and preparations	10.2
Other	1	Coffee	28.1	Coffee	19.2
	2	Wheat flour products	7.3	Orange juice	10.4
	3	Orange juice	5.1	Wheat flour products	5.1
Total polyphenols	1	Coffee	57.4	Coffee	49.8
	2	Bean and preparations	16.8	Bean and preparations	12.5
	3	Orange juice	4.5	Orange juice	5.9

The main food contributors to carotenoid intake, according to income level, are shown in Table 3. Pumpkin contribution to *α*-carotene, *β*-carotene and total carotenoids intake was greater in the lowest income group compared to the highest income. Corn and preparations consumption presented a higher contribution to lutein and zeaxanthin intake for individuals with lower income than higher income group. Salads had an important contribution to *α*-carotene, *β*-carotene, lutein, neoxanthin, violaxanthin and total carotenoids intake, especially in the highest income group. The contribution of fruits and vegetables in general, such as carrot, orange juice, papaya, tangerine, tomato, kale and orange to all carotenoid classes intake increased in the highest income group compared with the lowest income group.

**Table 3:** Comparison of the main food sources of carotenoids according to per capita income in Brazil (2017–2018).

Polyphenol classes and subclasses	Rank	Lowest per capita income		Highest per capita income	
		Food item	%	Food item	%
*α*-carotene	1	Salads	10.1	Salads	13.6
	2	Pumpkin	8.2	Pumpkin	6.4
	3	Carrot	4.4	Carrot	7.4
*β*-carotene	1	Pumpkin	11.9	Salads	13.1
	2	Salads	10.3	Pumpkin	7.8
	3	Carrot	3.9	Carrot	5.2
*β*-cryptoxanthin	1	Orange juice	14.5	Orange juice	16.0
	2	Papaya	6.7	Papaya	13.2
	3	Tangerine	4.1	Tangerine	8.4
Lycopene	1	Tomato	15.3	Tomato	27.3
	2	Tomato sauce	14.8	Tomato sauce	12.8
	3	Papaya	2.0	Papaya	5.3
Lutein	1	Salads	12.6	Salads	18.2
	2	Corn and preparations	8.4	Eggs	5.7
	3	Eggs	6.3	Corn and preparations	2.0
Neoxanthin	1	Salads	22.1	Salads	28.8
	2	Mango	4.5	Kale	5.4
	3	Kale	3.5	Mango	3.2
Violaxanthin	1	Salads	20.1	Salads	28.9
	2	Mango juice	8.7	Mango	5.9
	3	Mango	8.5	Mango juice	5.2
Zeaxanthin	1	Corn and preparations	38.8	Corn and preparations	15.1
	2	Orange juice	8.2	Orange juice	11.3
	3	Mango	4.4	Orange	4.2
Total carotenoids	1	Salads	9.5	Salads	15.0
	2	Pumpkin	6.3	Pumpkin	5.6
	3	Tomato	3.1	Tomato	5.3

Concentration indexes (CI) and coefficients of horizontal inequality (HI) indicate decrease in socioeconomic inequalities in favor of individuals with higher income regarding the consumption of flavonoids, other phenolic compounds, *β*-carotene, *β*-cryptoxanthin, lycopene and neoxanthin during the period of analysis (Table 4).

**Table 4:** Concentration indices for polyphenol classes intake. Brazil, 2008–2009 and 2017–2018.

Bioactive compounds	2008–2009		2017–2018		Differences	
	CI	HI	CI	HI	CI	HI
Polyphenols classes						
Phenolic acids	-0.05700	-0.06591	0.01757	0.01211	0.07457	0.07801
Flavonoids	0.10211	0.10211	0.07328	0.08524	-0.02883	-0.01686
Other polyphenols	0.08548	0.07541	0.04638	0.06803	-0.03910	-0.00739
Carotenoids classes						
*α*-carotene	0.03485	0.00496	0.04043	0.01699	0.00558	0.01203
*β*-carotene	0.09975	0.06622	0.08910	0.06702	-0.01065	0.00080
*β*-cryptoxanthin	0.10280	0.07390	0.08330	0.08355	-0.01950	0.00965
Lycopene	0.13885	0.11526	0.09020	0.09020	-0.04866	-0.02506
Lutein	0.07578	0.07604	0.08547	0.07203	0.00969	-0.00401
Neoxanthin	0.12680	0.10222	0.10460	0.09788	-0.02220	-0.00434
Violaxanthin	0.04161	0.04161	0.06371	0.04855	0.02211	0.00694
Zeaxanthin	-0.00970	0.00284	-0.02255	-0.02281	-0.01285	-0.02565

CI: concentration index, HI: horizontal index.

CI and HI in intake of phenolic acids was negative in 2008–2009, indicating inequality in favor of individuals with lower income; however, changed in favor of higher income individuals in 2017–2018. Therefore, trends in socioeconomic inequalities in consumption of phenolic acids showed transition towards individuals with higher income throughout time; yet, still showing lower degree of inequality (CI: 0.017571) compared to intake of flavonoids and other polyphenols (CI: 0.85244 and 0.068026, respectively).

The consumption of *α*-carotene, lutein and violaxanthin was concentrated among higher income individuals in 2008–2009, showing increase in socioeconomic inequality throughout the period. Zeaxanthin was the only carotenoid presenting concentration index in favor of lower income individuals in 2008–2009 and 2017–2018. In addition, considering changes in concentration index and horizontal inequality, there was increase in socioeconomic inequality favorable to poorer individuals in zeaxanthin intake, independently of individuals’ characteristics.

The analysis of factors influencing socioeconomic inequalities showed major influence of educational attainment on inequalities in consumption of flavonoids and other phenolic compounds in 2008–2009, whilst income was main factor of inequality in polyphenols intake during 2017–2018 (Fig 1). In relation to carotenoids, income was the main factor of inequality in lutein, neoxanthin and violaxanthin intake in 2008–2009, and its influence also encompassed lycopene, *β*-cryptoxanthin, *β*-carotene and *α*-carotene in 2017–2018 (Fig 2).

## Discussion

The present study explored ten-year trends in the associations between bioactive compounds intake and income in a representative sample of the Brazilian population. Our results indicated that dietary intake of polyphenols and carotenoids was significantly associated with income after adjustments for potential confounders, being higher income associated with higher intake of bioactive compounds.

The results of the present study are consistent with other data regarding the association between income level and bioactive compounds intake. Australian adolescents with higher income consumed more polyphenols than their counterparts (513 and 111 mg/d, respectively) [29]. US adults with high poverty-income ratio had significantly higher total flavonoids intake than low poverty-income ratio group (213.9 and 156.2 mg/d, respectively) [30], and Korean adults in the highest household income level had higher consumption of total flavonoid than those with lower household income (355.6 and 241.3 mg/d, respectively) [31].

Regarding the associations between income level and carotenoid intake, although data are still scarce, similar results were reported. Adults from NHANES [32] with higher income had greater total carotenoid intake than their counterparts (2.2 and 2.8 μg/ kg per day, respectively), and in the American cohort SELF, *β*-carotene and lycopene intake was positively associated with household income [33]. Socioeconomic status was also positively associated with plasma concentrations of lutein/zeaxanthin, lycopene, *α*- and *β*-carotene in the Australian cohort DRUID [34].

The findings support the body of evidence showing the substantial influence of socioeconomic status on diet quality, including in developing countries such as Brazil. Lower-income individuals usually consume diets with limited diversity of foods, fewer fruits and vegetables (i.e., lower in fiber content), and more energy-dense foods rich in fat, sugar and salt compared with higher-income individuals [35–41]. A major determinant of socioeconomic gradients in diet quality refers to higher cost of micronutrient-dense foods in comparison to energy-dense foods, which are often cheaper sources of calories [35–37]. In addition to lower intake of fruit and vegetables, which are main sources of polyphenols and carotenoids, individuals with limited resources tend to consume foods with high contents of fats, sugar and salt, and lower contents of fiber [36–38].

The disparities in eating patterns across income categories are also identified in the Brazilian population. Food intake data from NDS showed that the consumption of energy-dense foods (e.g., rice, cassava flour, corn, bread, pasta and sausages) is higher among low-income than high-income groups. In addition, individuals with lower income also presented lower per capita intake of fruits (e.g., orange, banana, apple, tangerine, papaya, mango, and grape) and vegetables (e.g., salads, lettuce, kale, cabbage, tomato, carrot, and cucumber) than their counterparts [23]. However, it is important to note that lower-income Brazilians also consume higher amounts of traditional foods (like rice, beans and corn) in comparison to higher-income individuals, which comprises a positive finding, supported by previous evidence [42].

The socioeconomic differences also support the variation in contributions of diverse food sources to bioactive compounds intake according to income. The decrease in phenolic acids and hydroxycinnamic acids intake across income categories in 2017–2018 compared to 2008–2009 is consistent with reduction in coffee intake during the period, which was more pronounced in the high-income group comparing with the low-income group. Similarly, the reduction in flavonoids and flavanones intake in 2017–2018 compared to 2008–2009 across the highest income group could be justified to the decrease in orange intake [23]. Regarding carotenoid intake, the greater consumption of carotenoids across income categories in 2017–2018 compared to 2008–2009 is consistent with higher intake of salad and vegetables in general, which is also more pronounced in the high-income group comparing with the low-income group [23].

Our results showed higher contribution of wine and tea to polyphenol intake according to income level, which is consistent with previous studies [4, 43]. The contribution of wheat flour products to intake of flavone and other polyphenols declines according to increase in income, which is supported by the lower prices of refined-grain foods in comparison with fresh and whole-grain foods [39, 44–46].

The contribution of corn and preparations to carotenoids intake decreased according to increase in income, potentially reflecting the nutritional transition process that Brazil is facing in the last 30 years, considering that comprise traditional foods consumed in some Brazilian regions (mainly North and Northeast regions). Besides the decrease in consumption of in natura and minimally processed foods and the increase in ultraprocessed food intake, the low-income groups tend to preserve some traditional eating habits, such as cereals and roots intake [47]. This could illustrate the greater contribution of corn and preparations to carotenoids intake among the low-income group compared with the highest income category.

Comparing inequalities in polyphenol intake between 2008–2009 and 2017–2018, our results indicate reduction in socioeconomic inequality towards richer individuals in consumption of certain polyphenols (flavonoids and other polyphenols) and carotenoids (*β*-carotene, *β*-cryptoxanthin, lycopene, and neoxanthin) during the period. Yet, the disaggregation of concentration indexes suggests that income still represents the main barrier to polyphenol and carotenoid intake in 2017–2018. The finding corroborates concerns regarding the role of socioeconomic patterns in determining food consumption patterns and, thus, healthy eating [4, 17–22, 48].

Furthermore, we point out the potential of many regional fruits and vegetables to increase bioactive compounds intake in lower income groups. Additionally, the publication of the polyphenol and carotenoid composition data of regional foods without a described content, such as abricó, bacabá, babaçu, bertalha, cajá-manga, ciriguela, fruta-pão, jambo, jurubeba and pitomba, can be an important economic strategy to be explored. The promotion of local utilization of these regional foods, as well as the development of new marketable local products, can play a major role in strengthening rural development, creating business opportunities, and reducing poverty. Finally, this scenario could contribute to reinforce agrifood systems and the sustainable development of agriculture [49].

The present study has several strengths. First, we use nationally representative sample of the Brazilian population, adopting complex sampling design, allowing generalization of results at population level. Second, the use of Phenol-Explorer data, which is the most comprehensive database of polyphenols, combined with information on Brazilian food composition obtained by high-quality analytical methods, increase the robustness of study estimates. Third, the identification of socioeconomic inequalities in polyphenol intake is useful for establishing public health policies towards healthy food consumption patterns. However, the present study has also limitations. First, the two NDS editions collected information on food consumption through different instruments, although results remained mostly comparable. Second, the 2008–2009 and 2017–2018 NDS present cross-sectional design, thus, it is not possible to establish causality in polyphenol intake. Nonetheless, it is important to emphasize that the adoption of the standard procedures in the analysis of the two NDS tend to minimize potential bias in the results of the study. Third, the consumption of polyphenols and carotenoids was based on estimation, which may not reflect the exact intake. Other limitations are the lack of information of polyphenol and carotenoid composition in some local fruits and vegetables consumed in Brazil, as well as the lack of information on the content of polyphenol and carotenoid in cooked foods.

## Conclusion

In conclusion, the present study showed that income level is an important determinant of bioactive compounds intake in the Brazilian population throughout the period between 2008–2009 and 2017–2018, being the main factor associated with socioeconomic inequalities in polyphenols and carotenoids intake in 2017–2018. The main food sources contributing to polyphenols and carotenoids intake according to income corroborates evidence on disparities related to socioeconomic patterning in eating choices, showing greater contribution of fruits and vegetables to bioactive compounds intake according to increase in income. We highlight the importance of future studies investigating associations between bioactive compounds intake and chronic diseases risk, specially between low-income individuals. Also, our results may encourage the monitoring of polyphenols and carotenoids intake among different income groups, information that may be useful for identification of potential intervention targets and for improvement in effectiveness of public health interventions towards healthy food consumption patterns in the Brazilian population.
